# Monkeypox virus H3L protein as the target antigen for developing neutralizing antibody and serological assay

**DOI:** 10.1007/s00253-025-13466-6

**Published:** 2025-04-02

**Authors:** I-Hsiang Huang, Guan-Chun Lai, Tai-Ling Chao, Wang-Da Liu, Sui-Yuan Chang, Shih-Chung Chang

**Affiliations:** 1 Department of Biochemical Science and Technology, College of Life Science, National Taiwan University, Taipei, 106 Taiwan; 2 Department of Clinical Laboratory Sciences and Medical Biotechnology, College of Medicine, National Taiwan University, Taipei, 100 Taiwan; 3 Department of Internal Medicine, National Taiwan University Hospital, College of Medicine, National Taiwan University, Taipei, 100 Taiwan; 4 Department of Medicine, National Taiwan University Cancer Center, Taipei , 106 Taiwan; 5 Department of Laboratory Medicine, National Taiwan University Hospital, College of Medicine, National Taiwan University, Taipei, 100 Taiwan; 6 Center of Biotechnology, National Taiwan University, Taipei, 106 Taiwan

**Keywords:** Monkeypox virus (MPXV), H3L protein, Lateral flow immunochromatographic assay, Neutralizing antibody, Complement, Serological assay

## Abstract

**Abstract:**

The large number of atypical monkeypox (Mpox) cases caused by emerging monkeypox virus (MPXV) strains was recently found in countries and regions where the Mpox was not reported before. Diagnostic tools and therapeutic agents are important countermeasures for preventing Mpox outbreak. H3L protein is the important surface antigen of MPXV for binding to host cell receptors and mediating viral infection. A broad range of murine anti-MPXV H3L monoclonal antibodies (mAbs) recognizing various binding epitopes have been generated in the study. The rapid test composed of the mAbs 4-2A and 3-3F can specifically detect H3L protein and MPXV virion. The mAb 3-3F exhibited strong MPXV neutralizing activity in a complement-dependent manner. Notably, 3-3F binds to a unique epitope within residues 35–89 of H3L protein. The serum samples collected from Mpox patients barely bound to the N-terminal portion of H3L protein ranging from 2 to 89 residues, indicating that the content of the 3-3F-like antibody is very low in Mpox patient sera. In contrast, the seropositivity was mostly observed using the C-terminal portion of H3L protein ranging from 185 to 282 residues as the target antigen in the immunoblot analysis. Taken together, the anti-MPXV H3L mAb can be developed as the Mpox diagnostic and therapeutic agents. Furthermore, H3L protein is the promising biomarker for serological analysis.

**Key Points:**

•*Anti-H3L mAbs can cross-react with H3L proteins in MPXV and VACV virions.*

•*The LFIA rapid test using the mAbs 4-2A and 3-3F can specifically detect MPXV.*

•*MPXV was neutralized by mAb 3-3F in a complement-dependent manner*

## Introduction

Mpox (formerly known as monkeypox) is a viral disease caused by an orthopoxvirus, monkeypox virus (MPXV), and has been reported in Africa for several decades since it was first detected in humans in 1970 (Foster et al. 1972; Ladnyj et al. 1972). MPXV shares similar clinical features with smallpox and is considered the most severe orthopoxvirus infection since the eradication of smallpox (Gong et al. 2022). MPXV is divided into two major clades: clades I and II, respectively (Van Dijck et al. 2023). The fatality rate for clade I is as high as 10%, while it is less than 1% for clade II (Mitja et al. 2023). From July 2022 to August 2024, the global outbreak of Mpox was declared a public health emergency of international concern (PHEIC) for two times by World Health Organization (WHO) as it spread rapidly across a range of countries and regions where the virus had not been previously reported (Haque et al. 2024; Rizk et al. 2024). Notably, the upsurge of Mpox with the large number of atypical cases was caused by the emerging virus strains (Gigante et al. 2022; Isidro et al. 2022; Otieno et al. 2024; Zhu et al. 2023) with different modes of transmission and various levels of risk (Cho et al. 2024; Okwor et al. 2023; Rampogu et al. 2023; Saraswat and Shah 2024; Sukhdeo et al. 2022). The multi-country outbreak of Mpox was a shocking warning and should not be neglected (Haque et al. 2024). In response to the Mpox outbreak, it is time to act decisively to develop the specific diagnosis tools, effective vaccines, and therapeutic agents.

The process of MPXV infection can be summarized into three distinct stages: virus invasion, replication, and release (Lu et al. 2023). If the MPXV invasion is blocked, there is no subsequent virus life cycle undergoing inside the host cell. Therefore, the surface membrane proteins of MPXV involved in binding to human membrane receptors become important targets for neutralizing antibody (Hubert et al. 2023; Yefet et al. 2023; Zeng et al. 2023). It has been shown that vaccinia virus (VACV) H3L, sharing 93.52% sequence similarity with MPXV H3L (Sagdat et al. 2024), binds heparin sulfate (Lin et al. 2000; Singh et al. 2016) but does not bind well to human cells that are deficient in surface glycosaminoglycans (Singh et al. 2016). These observations imply that H3L plays the crucial role in the viral adsorption to the host cells. Additionally, the absence of H3L disrupts the assembly of intracellular mature virions (MV), indicating that H3L is also involved in the transformation and assembly of viral particles (Lin et al. 2000). Many lines of evidence indicated that anti-H3L antibodies exhibited virus neutralizing capabilities. It has been reported that the immunization of MPXV A29L and H3L antigens in mice can induce the highest neutralization titers of sera against authentic MPXV (Song et al. 2024). Human anti-VACV H3L mAb hV26 has been demonstrated with therapeutic efficacy against a lethal VACV infection in the immunodeficiency mice (Crickard et al. 2012; McCausland et al. 2010). An anti-H3L mAb MV32, selected from the phage-display library which was constructed based on the macaques immunized with live VACV, exhibited high affinity and potent in vitro VACV neutralization capabilities. Furthermore, mAb MV32 was also able to neutralize Mpox 2018 and 2022 strains, suggesting a potential for cross-poxvirus protection (Noy-Porat et al. 2023).

The abundant surface antigens of MPXV are also specific marker proteins for use in diagnostics and serological assays (Cohn et al. 2023; Liang et al. 2024; Yang et al. 2023). H3L has been identified as one of the major immunogenic proteins of VACV, which was capable of inducing both T-cell and B-cell immune responses (Drexler et al. 2003; Ostrout et al. 2007). H3L is also considered the immune target after MPXV infection (Yefet et al. 2023). Sera from Mpox convalescent patients bound strongly to H3L antigen (Yefet et al. 2023). In the serological study of Mpox patients, it was found that the positive rates of serum recognition of H3L could exceeded 80% within the first week of infection (Yefet et al. 2023), indicating that H3L could serve as the early diagnostic marker (Yang et al. 2024).

In the rapidly evolving viral landscape of Mpox, rapid detection and interruption of viral transmission chains are crucial strategies to prevent outbreaks. In the study, the recombinant MPXV H3L protein fused with an N-terminal maltose-binding protein (MBP) tag was purified from the *Escherichia coli* expression system in the soluble condition and used to immunize BALB/c mice for generating monoclonal antibodies (mAbs). Six anti-MPXV H3L mAbs, 4-2A, 1-1C, 3-8H, 3-3F, 4-9F, and 2-7G, were produced and further subjected to characterization of their isotypes, binding epitopes, and cross-reactivity with the VACV virions. The mAb pairing composed of 4-2A and 3-3F was developed as the lateral flow immunochromatographic assay (LFIA) for specific detection of the recombinant MPXV H3L proteins and the MPXV virions. The plaque reduction and neutralization test (PRNT) assays were performed to access their ability of neutralizing MPXV infection to Vero E6 cells (a cell line exhibiting epithelial morphology that was isolated from the kidney of an African green monkey) in the absence or presence of the baby rabbit complements. In addition, the truncated fragments of the recombinant MPXV H3L were applied in the serological assays to analyze the antibody profiles of the serum samples collected from Mpox patients. This study provided useful insights on using MPXV H3L protein as the promising target for development of the diagnostic and neutralizing antibody.

## Materials and methods

### Preparation of the recombinant MPXV H3L protein

The cDNA encoding for the extracellular portion of the MPXV H3L from amino acids 1–278 (GenBank accession number: WEY18844.1) was subcloned into the pET28a vector (Merck Millipore, Burlington, MA, USA) with the sequences for an N-terminal hexa-histidine tag (His-tag; MGSSHHHHHH), a linker peptide (SSGLVPRGSH), and a maltose-binding protein (MBP) tag (hereinafter named as pET28a-MBP-H3L). To construct the plasmids for expressing the truncated H3L fragments, the cDNA of the full-length H3L in the pET28a-MBP-H3L plasmid was replaced with the cDNA encoding for the H3L fragment 2–34, 2–89, 2–184, or 2–239, respectively. The *E. coli* Rosetta(DE3) competent cells were transformed with the plasmids (Merck Millipore, Burlington, MA, USA) in the presence of kanamycin (50 μg/mL) and chloramphenicol (34 μg/mL). For protein expression, bacteria were cultured in the Luria–Bertani (LB) medium to an OD_600_ of 0.5 at 37 °C and then induced with 1 mM isopropyl β-D-1-thiogalactopyranoside (IPTG) at 18 °C for 12 h. The bacterial lysate resuspended in the nickel-nitrilotriacetic acid (Ni–NTA) column binding buffer (20 mM sodium phosphate, 0.5 M NaCl, 10 mM imidazole, pH 7.4) was loaded on the HisTrap column (Cytiva, Marlborough, MA, USA), and the bound proteins were eluted with a 10–250 mM imidazole gradient. The collected fractions were exchanged buffer with 20 mM Tris–HCl (pH 7.4), 200 mM NaCl, 1 mM ethylenediaminetetraacetic acid (EDTA), and 1 mM dithiothreitol (DTT) by using the PD-10 column (Cytiva, Marlborough, MA, USA). Samples were then loaded on the MBPTrap column (Cytiva, Marlborough, MA, USA) and eluted with 10 mM maltose. The purified recombinant MBP-H3L proteins were exchanged buffer with phosphate-buffered saline (PBS) by using the PD-10 column and stored at − 20°C before use.

### Generation of mouse mAb

The procedures for immunization of antigen and generation of hybridomas were performed as described previously with slight modifications (Cheng and Chang 2021; Lai et al. 2022; Li et al. 2022). In brief, 7-week-old BALB/c mice were purchased from the National Laboratory Animal Center Taiwan and immunized four times in a 2-week interval with 50 µg of MBP-H3L formulated with 50 µL of Freund’s adjuvant (EMD Millipore Corporation, Burlington, MA, USA) through intraperitoneal injection. Seven days post each immunization, mouse antisera were collected from the submandibular veins for analysis of the elicited antibody levels. Three days post the final booster with 50 µg of MBP-H3L in PBS, mice were sacrificed in accordance with the animal care and ethics guidelines. Subsequently, splenocytes were mixed with Sp2/0-Ag14 cells (ATCC CRL-1581) in the presence of polyethylene glycol 1500 (Sigma-Aldrich, USA) for performing the cell fusion step. Cells were resuspended in Dulbecco’s Modified Eagle Medium (DMEM) containing 15% fetal bovine serum, 1% penicillin–streptomycin, 1 mM sodium pyruvate, and hypoxanthine-aminopterin-thymidine (HAT) supplement (EMD Millipore Corporation, Burlington, MA, USA) and aliquoted into 96-well cell culture plates at 37 °C in the 5% CO_2_ incubator. Seven days post cell fusion, hypoxanthine-thymidine (HT) supplement (EMD Millipore Corporation, Burlington, MA, USA) was added to each well for improving cell growth. The culture media were collected for enzyme-linked immunosorbent assay (ELISA) using 100 ng of the purified recombinant MBP-H3L protein as the antigen. The positive hybridoma cells were selected by limiting dilution method.

### Western blot (WB) analysis

Proteins or viral samples were analyzed on the sodium dodecyl sulfate–polyacrylamide gel electrophoresis (SDS-PAGE) under the reduction condition. Protein bands were transferred to the polyvinylidene difluoride (PVDF) membranes (EMD Millipore Corporation, Burlington, MA, USA), which were then blocked with 5% non-fat milk in PBS containing 0.05% Tween 20 (PBST) at room temperature for 30 min. The mouse antisera or the mAb samples (1:5000 in blocking buffer) were used to detect the target antigens at room temperature for 1 h. After washing steps with PBST, the horseradish peroxidase (HRP)-conjugated secondary antibody (5450–0011, SeraCare, Milford, MA, USA) was used for probing the primary antibody. The WB signal was developed by using the chemiluminescent substrate (VisGlow, Visual Protein Biotechnology, Taipei, Taiwan) and captured by the UVP BioSpectrum Imaging System (Upland, CA, USA).

### Purification of mAb

The culture media of the hybridoma cells were loaded on the HiTrap Protein G HP column (Cytiva, Marlborough, MA, USA) which was pre-equilibrated with 20 mM sodium phosphate (pH 7.0). Bound antibody samples were eluted with 0.1 M glycine–HCl buffer (pH 2.7) and immediately neutralized to pH 7.4 with 1 M Tris–HCl (pH 9.0). The isotypes of the purified mAbs were determined by using the Rapid ELISA Mouse mAb Isotyping Kit (Thermo Fisher Scientific, Waltham, MA, USA) according to the manufacturer’s instruction. The purified mAbs were exchanged buffer with PBS by using the PD-10 column (Cytiva, Marlborough, MA, USA) and then stored at − 20 °C before use.

### PRNT assay

Approximately 2 × 10^5^ Vero E6 cells (ATCC CRL-1586) were seeded in each well of the 24-well plate and cultured in DMEM supplemented with 10% FBS and antibiotics for 1 day at 37 °C in the 5% CO_2_ incubator. On the day for performing the viral infection step, MPXV (50 PFU) was mixed with antibody sample in the absence or presence of 10% baby rabbit complements (31,061–1, Pel-Freez, Rogers, AR, USA) at 37 °C for 1 h. The mixture was then added to the Vero E6 cell monolayer seeded in the well of the 24-well plate and incubated at 37 °C for 1 h. After that, the mixture was removed and an overlay medium was added, followed by 7 days of incubation. The cell monolayer was then fixed with 10% formalin (equivalent to 3.7% formaldehyde) overnight. After the removal of formalin, the cells were stained with 0.5% crystal violet to visualize the plaque formation.

### LFIA

The anti-MPXV H3L mAb 3-3F (60 μg/mL) was mixed with 40 nm colloidal gold (O.D.520 = 1, DCN, Carlsbad, CA) in 20 mM borate buffer (pH 9.0) at 4 °C overnight and then blocked with 10% bovine serum albumin (BSA). The 3-3F-conjugated colloidal gold was collected by centrifugation at 7000 rpm for 15 min and then resuspended in 20 mM Tris–HCl (pH 8.2) containing 0.05% polyethylene glycol (PEG) 20,000, 150 mM NaCl, 1% BSA, and 0.1% sodium azide. Conjugate pad (Cytiva, Marlborough, MA, USA) was pre-treated with 20 mM Tris–HCl (pH 8.2) containing 1% BSA, 6% trehalose, 4% sucrose, and 0.05% sodium azide and then coated with the 3-3F-conjugated colloidal gold solution. The capture mAb 4-2A, 1-1C, or 3-8H was coated in the test line, and protein G was coated in the control line of the nitrocellulose (NC) membrane (UniSart CN140, Sartorius, Göttingen, Germany). The NC membrane was then thoroughly blocked with 0.5% casein in 50 mM borate buffer (pH 8.5) at room temperature for 30 min and washed with 0.5% sucrose in 50 mM Tris–HCl (pH 7.5). After that, the NC membrane was air-dried at 50 °C for 30 min and subsequently assembled with sample pad, adsorbent pad, and the rapid test cassette. The recombinant MBP-H3L proteins or the MPXV virions were mixed with 75 $$$L of lysis buffer (50 mM Tris, 1% BSA, 0.2 M NaCl, 0.05% Triton X-100) to perform the LFIA at room temperature for 5–10 min.

## Results

### Immunization of MPXV H3L in BALB/c mice

**Fig. 1:**
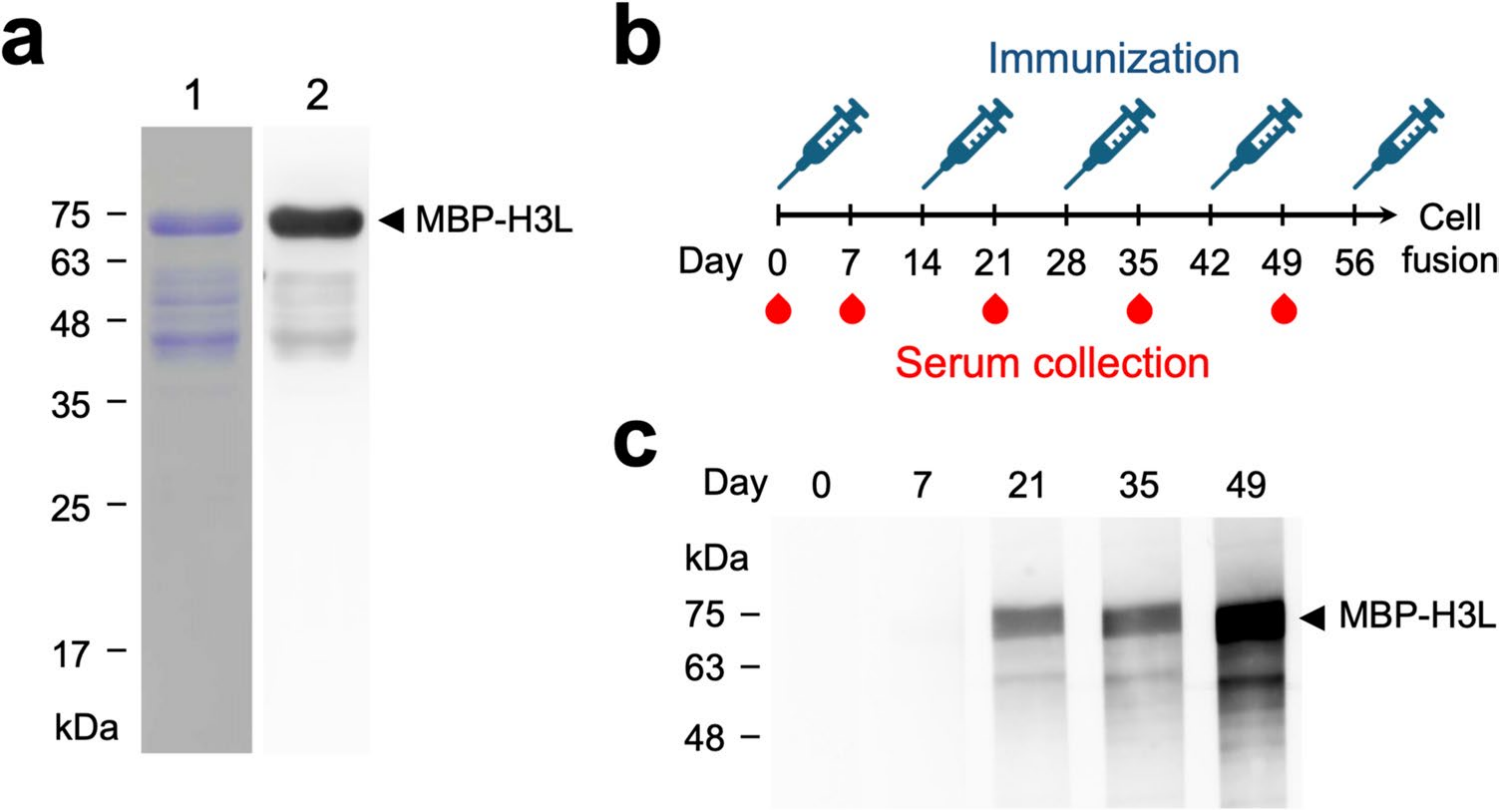
Immunization of mice with the recombinant MPXV MBP-H3L protein. **a** The purified MBP-H3L was analyzed on SDS-PAGE, coomassie staining (left panel), and WB with anti-His tag antibody (right panel). **b** BALB/c mice were immunized with five doses of the purified MBP-H3L in a 2-week interval. The mouse antisera were collected five times at days 0 (before immunization), 7, 21, 35, and 49 as indicated with red blood symbols in the diagram. **c** The collected mouse antisera were applied in the WB for monitoring the induction of the MBP-H3L-specific antibodies throughout the prime-boost immunization process

In order to produce the recombinant MPXV MBP-H3L proteins for immunization of BALB/c mice, the *E. coli* Rosetta(DE3) competent cells were transformed with the pET28a-MBP-H3L plasmid for protein expression, and the cell lysates were further loaded on the HisTrap and MBPTrap columns for protein purification as described previously. The purified MBP-H3L protein was subjected to the SDS-PAGE and the WB analysis (Fig. 1a). The BALB/c mice were immunized in a 2-week interval with four doses of MBP-H3L proteins supplemented with the Freund’s adjuvant and a final booster of MBP-H3L protein solution in PBS (Fig. 1b). The mouse antisera were collected 1 week post first to fourth immunization and then used to measure the immune response against MBP-H3L by WB analysis. The results showed that the MBP-H3L-specific antibodies were significantly elicited in the antiserum collected on day 49 (Fig. 1c).

### Production of the H3L-specific mAbs

**Fig. 2:**
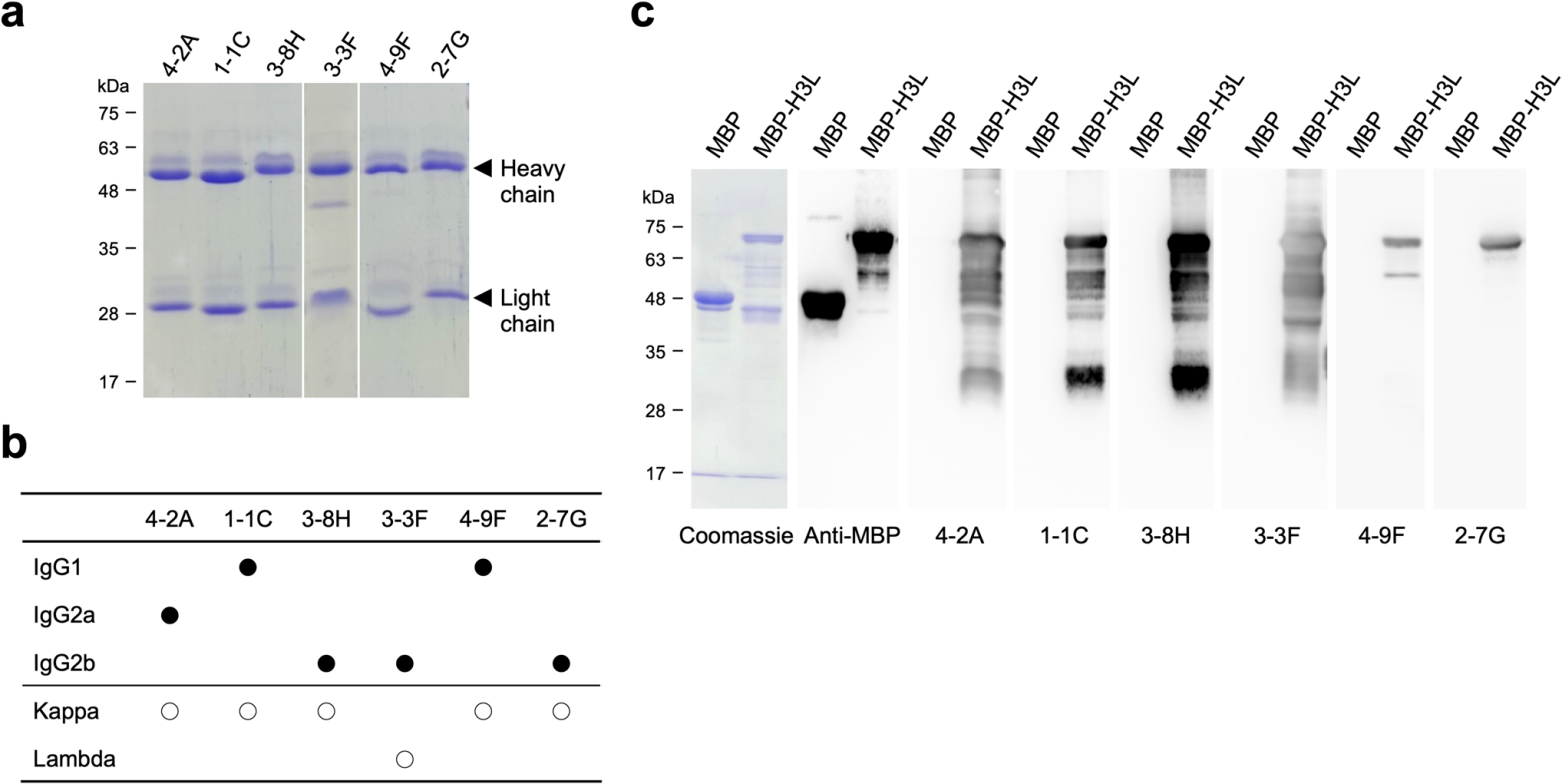
Characterization of the anti-H3L mAbs. **a** The purified 4-2A, 1-1C, 3-8H, 3-3F, 4-9F, and 2-7G were analyzed on SDS-PAGE under the reduction condition and further revealed by coomassie staining. **b** The isotypes of the anti-H3L mAbs were determined by using the Rapid ELISA Mouse mAb Isotyping Kit according to the manufacturer’s instruction. The isotypes of the heavy chains and light chains for each mAbs were indicated with solid and open circles, respectively. **c** The specificity of the anti-H3L mAbs was identified by WB analysis using MBP and MPB-H3L as the antigens. Anti-MBP was used as the control antibody to detect both of MBP and MBP-H3L

The splenocytes obtained from the immunized BALB/c mice were fused with Sp2/0-Ag14 cells to generate the hybridoma cell lines. Six mAbs against MPXV H3L were generated in the study, including 4-2A, 1-1C, 3-8H, 3-3F, 4-9F, and 2-7G (Fig. 2a). The isotypes of the purified mAbs were also determined by using the Rapid ELISA Mouse mAb Isotyping Kit. The results revealed the heavy chain and light chain isotypes of these mAbs, including IgG2a/kappa (4-2A), IgG1/kappa (1-1C and 4-9F), IgG2b/kappa (3-8H and 2-7G), and IgG2b/lambda (3-3F), respectively (Fig. 2b). In addition, these six mAbs exhibited great specificity against H3L without cross-reacting with MBP (Fig. 2c). Notably, the detection profiles of these six mAbs in the WB seemed to have at least three different patterns. 4-2A, 1-1C, 3-8H, and 3-3F detected multiple degraded MBP-H3L bands. 4-9F detected two bands, including the full-length MBP-H3L and one degraded fragment. In contrast, 2-7G only detected the full-length MBP-H3L (Fig. 2c).

### Characterization of the binding epitopes of anti-H3L mAbs

**Fig. 3:**
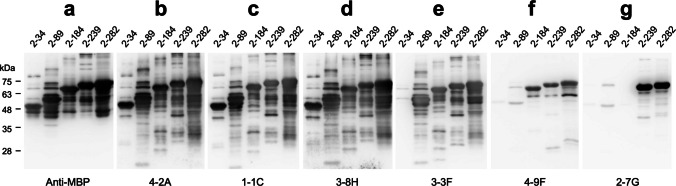
Characterization of the binding epitopes of anti-H3L mAbs. The truncated H3L fragments (2–34, 2–89, 2–184, 2–239, and 2–282) fused with an N-terminal MBP-tag were analyzed on SDS-PAGE and subjected to WB analysis with anti-MBP (**a**), 4-2A (**b**), 1-1C (**c)**, 3-8H (**d**), 3-3F (**e**), 4-9F (**f**), and 2-7G (**g**), respectively

To further determine the binding epitopes of these six anti-H3L mAbs, a series of truncated MBP-H3L fragments were separated on SDS-PAGE and subjected to WB analysis with the indicated antibodies: anti-MBP antibody, 4-2A, 1-1C, 3-8H, 3-3F, 4-9F, and 2-7G, respectively (Fig. 3a–g). The results showed that 4-2A, 1-1C, and 3-8H recognize all of the truncated MBP-H3L fragments (Fig. 3b–d), suggesting that the binding fragments of these three mAbs are located within residues 2–34 of H3L protein. 3-3F can bind to all of the truncated MBP-H3L fragments, except the MBP-H3L_2-34 (Fig. 3e), suggesting that the binding fragment of 3-3F is located within residues 35–89 of H3L protein. 4-9F recognize the truncated MBP-H3L fragments larger than MBP-H3L_2-184 but cannot bind to MBP-H3L_2-34 and MBP-H3L_2-89 (Fig. 3f), indicating that the binding fragment of 4-9F is located within residues 90–184 of H3L protein. Furthermore, 2-7G only binds to MBP-H3L_2-239 and MBP-H3L_2-282 but not MBP-H3L_2-34, MBP-H3L_2-89, and MBP-H3L_2-184 (Fig. 3g), indicating that the binding fragment of 2-7G is located within residues 185–239 of H3L protein. Collectively, these six anti-H3L mAbs bind to H3L protein and truncated fragments through recognizing at least four different epitopes.

### Anti-H3L mAbs cross-reacted with MPXV and VACV virions

**Fig. 4:**
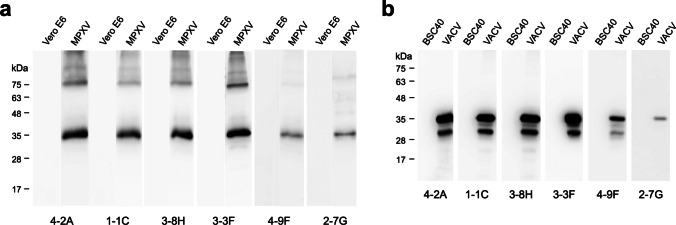
Recognition of the authentic H3L proteins in MPXV and VACV virions by anti-H3L mAbs. **a** The culture media collected from the control Vero E6 cells or the MPXV-infected Vero E6 cells, which produced MPXV virions, were analyzed on SDS-PAGE and subjected to WB analysis with 4-2A, 1-1C, 3-8H, 3-3F, 4-9F, or 2-7G, respectively. **b** The culture media collected from the control BSC40 cells or the VACV-infected BSC40 cells, which produced VACV virions, were analyzed on SDS-PAGE and subjected to WB analysis with 4-2A, 1-1C, 3-8H, 3-3F, 4-9F, or 2-7G, respectively

The MPXV and VACV H3L proteins share 93.52% sequence similarity (Sagdat et al. 2024). Therefore, we also examined whether the anti-H3L mAbs produced in the study can recognize the authentic H3L proteins in the MPXV and VACV virions. The WB analysis results showed that all of the mAbs 4-2A, 1-1C, 3-8H, 3-3F, 4-9F, and 2-7G can cross-react with H3L proteins in MPXV and VACV virions (Fig. 4). It is also noted that the highest WB signal was observed by using 3-3F as the probing antibody (Fig. 4). In contrast, 4-9F and 2-7G exhibited significantly lower binding affinity against MPXV and VACV H3L proteins than other mAbs (Fig. 4).

### Detection of H3L protein and MPXV virion by LFIA

**Fig. 5:**
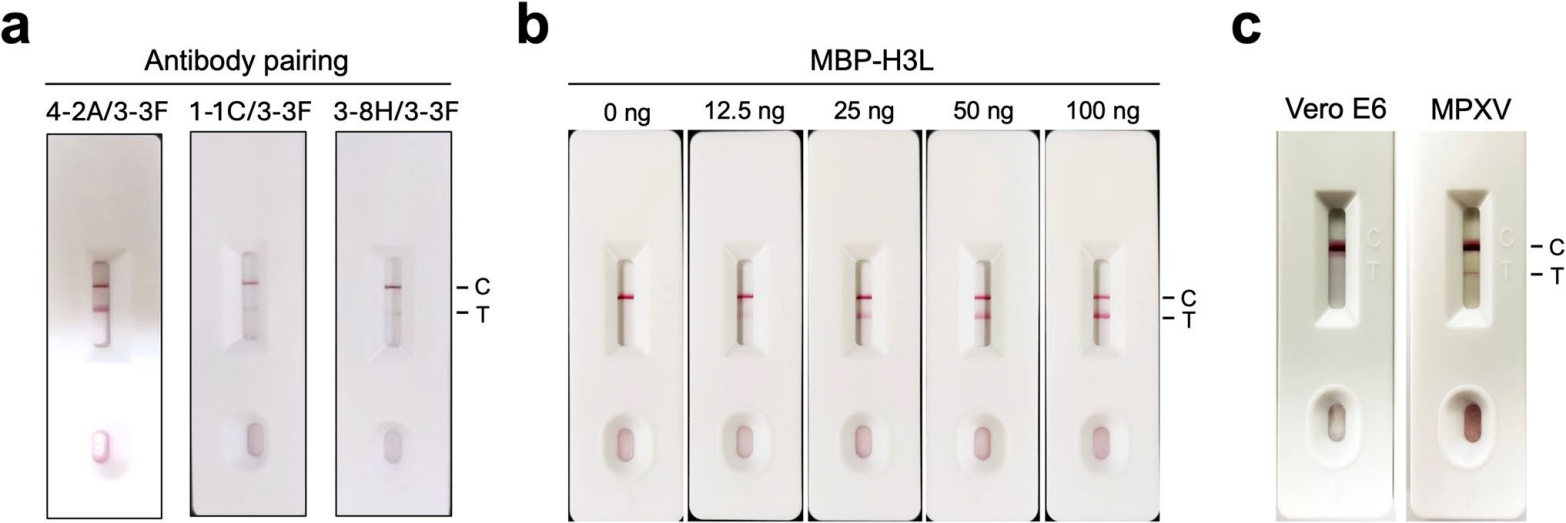
The anti-H3L mAb-based LFIA rapid test. **a** The mAb pairing 4-2A/3-3F, 1-1C/3-3F, or 3-8H/3-3F-based LFIA rapid test was assembled for evaluation of the binding efficiency for MBP-H3L. C, control line. T, test line. **b** The various amounts of MBP-H3L protein (0, 12.5, 25, 50, and 100 ng) were added to the 4-2A/3-3F-based LFIA rapid tests for determining the values of the limit of detection (LOD). **c** Vero E6 cell lysates and MPXV virions are also used to examine the detection specificity of the 4-2A/3-3F-based LFIA rapid test

As described previously, 3-3F is the best detection antibody for probing MPXV and VACV virions, but 4-9F and 2-7G poorly bind to them (Fig. 4). In addition, 4-2A, 1-1C, and 3-8H bind to a similar epitope which is different from the one recognized by 3-3F. Therefore, the mAb pairing composed of 4-2A/3-3F, 1-1C/3-3F, or 3-8H/3-3F was developed as the LFIA rapid test for evaluating the binding effectiveness against H3L protein. The experimental result showed that a clear detection signal appeared on the test line when MBP-H3L was added to the 4-2A/3-3F-based LFIA rapid test (Fig. 5a). Interestingly, there was no clear detection signal observed when MBP-H3L was added to the 1-1C/3-3F-based or 3-8H/3-3F-based LFIA rapid tests (Fig. 5a). To further determine the limit of detection (LOD) of the 4-2A/3-3F-based LFIA rapid test, the different amounts of MBP-H3L were applied in the experiments. The results showed that the minimum concentration that can be detected by the 4-2A/3-3F-based LFIA rapid test is 12.5 ng (Fig. 5b). In addition, the 4-2A/3-3F-based LFIA rapid test can capture the MPXV virions in the test line but not the Vero E6 control sample (Fig. 5c).

### Inhibition of MPXV infection by mAb 3-3F

**Fig. 6:**
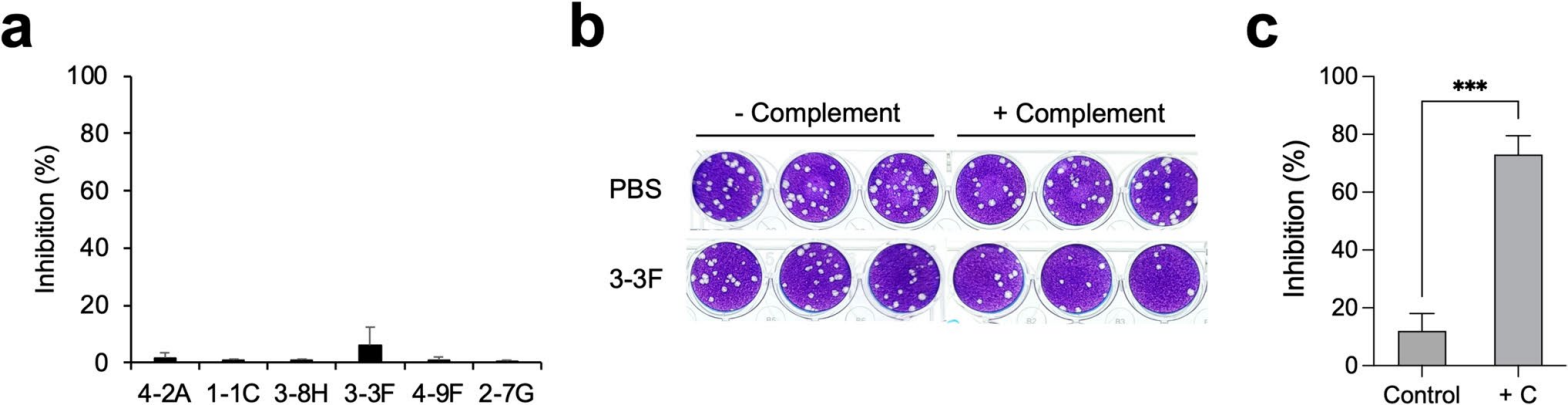
Neutralization of MPXV by 3-3F in the presence of complements. **a** The MPXV virions were pre-incubated with PBS, 4-2A, 1-1C, 3-8H, 3-3F, 4-9F, or 2-7G (50 μg/mL) and then added to Vero E6 cell monolayers for performing the PRNT assays, respectively. Percentage inhibition = [(control − antibody)/control] × 100. Control stands for the counting numbers of plaques formed in the PRNT assays with PBS. Antibody stands for the counting numbers of plaques formed in the PRNT assays with the indicated mAb. Data are presented as means ± SD of three biological replicates (*n* = 3). **b** The MPXV virions were pre-incubated with PBS or 3-3F (50 μg/mL) in the absence or presence of 10% baby rabbit complements (-/ + complement) and then added to Vero E6 cell monolayers for performing the PRNT assays. After incubation for 7 days, cells were fixed with formaldehyde and stained with crystal violet. Three biological replicates were conducted for each experimental group. **c** The counting numbers of plaques derived from **b** were calculated by using the formula described previously and presented as percentage inhibition. Control, without 10% baby rabbit complements. + C, with 10% baby rabbit complements

To investigate the neutralizing potential of these six anti-H3L mAbs, antibody samples (50 μg/mL) were pre-incubated with MPXV virions, and the mixtures were added to the Vero E6 monolayers for performing the PRNT assays. Notably, only 3-3F exhibited minor neutralizing activity (~ 12% inhibition) against MPXV infection to Vero E6 cells (Fig. 6a). Previous studies have suggested that complements may enhance the neutralizing activity of anti-VACV and anti-MPXV antibodies (Benhnia et al. 2009b; Hubert et al. 2023; Kaever et al. 2016; Lustig et al. 2005). Thus, we further examined whether the neutralizing activity of 3-3F can be enhanced in the presence of baby rabbit complements. The results of PRNT assays showed that the formation of viral plaques was profoundly decreased in the presence of 3-3F and baby rabbit complements (Fig. 6b). The results obtained from three independent experiments revealed that the neutralizing activity of 3-3F at the concentration of 50 μg/mL was significantly promoted from ~ 15% inhibition to ~ 75% inhibition of MPXV infection to Vero E6 cells in the presence of 10% baby rabbit complements (Fig. 6c).

### The antibody profiles of Mpox patient sera against different H3L domains

**Fig. 7:**
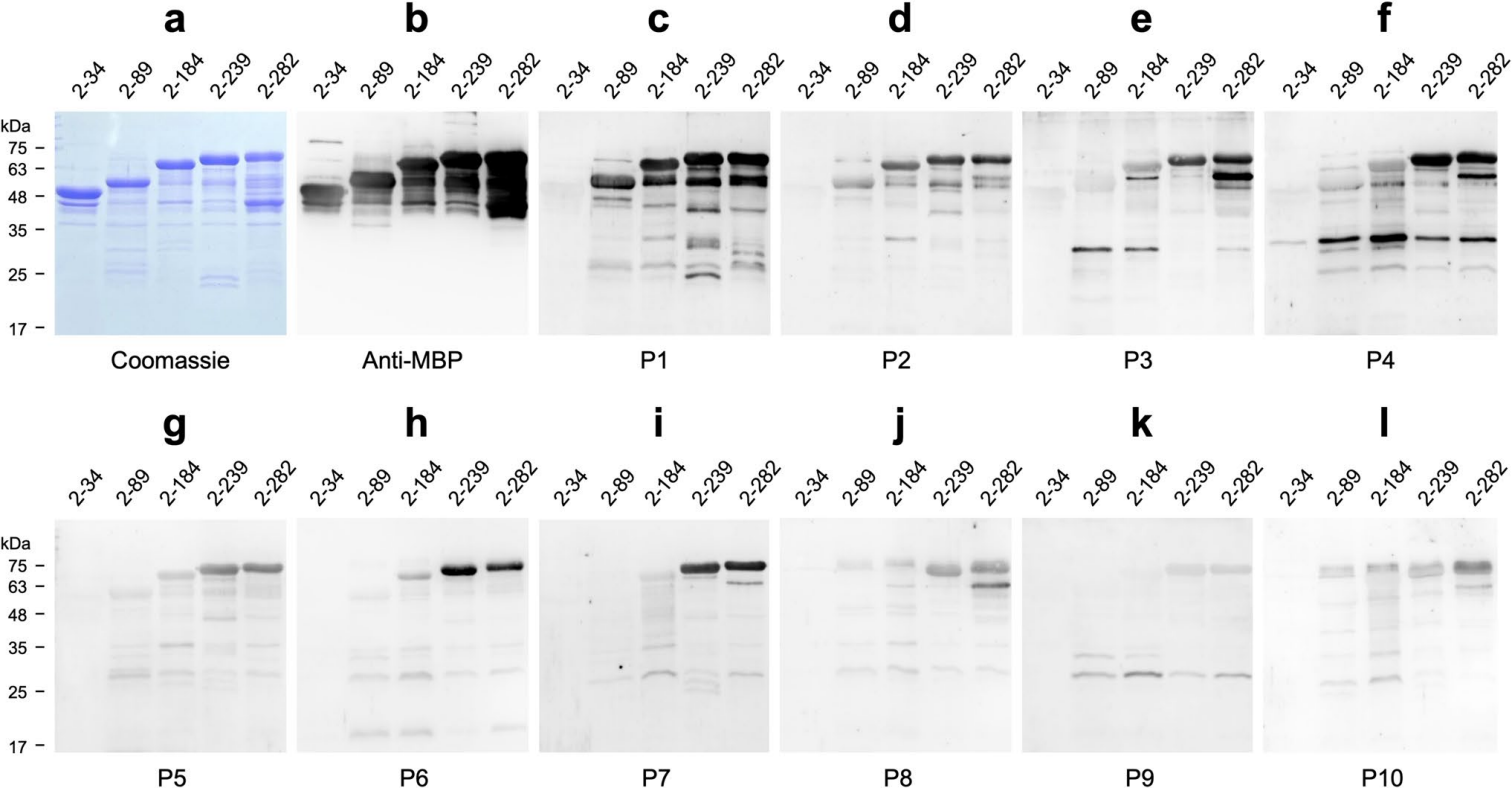
The antibody profiles of the Mpox patient sera against the truncated H3L fragments. The different lengths of the truncated H3L fragments (2–34, 2–89, 2–184, 2–239, and 2–282) fused with an N-terminal MBP-tag were analyzed on SDS-PAGE and subjected to WB analysis with anti-MBP (**a**) or the human sera (1:1000) collected from Mpox patients P1-10 (**c**–**l**) 2 weeks after admission to the hospital, respectively

H3L-specific antibody has been considered a target for diagnosis of MPXV infection in the serological assay (Yang et al. 2024; Yefet et al. 2023). However, the antibody profiles of Mpox patient sera against H3L protein have not been characterized. Furthermore, it remains uncertain whether the human immune system can generate antibodies targeting different domains of H3L protein. To address these issues, sera randomly collected from ten Mpox patients who have not received the smallpox vaccine were used to perform the WB analysis against a series of truncated H3L fragments which were previously utilized in the epitope mapping experiments (Fig. 7a & b). The results showed that the sera collected from all of the Mpox patients barely bound to the truncated peptide 2–34 (Fig. 7c–i), indicating that the H3L_2-34-specific antibodies were not responsively induced after natural infection by MPXV. Two Mpox patient sera can nicely bind to H3L_2-89 and other longer fragments (Fig. 7c & d). Five Mpox patient sera exhibited weaker binding against H3L_2-184, but they exhibited stronger binging against H3L_2-239 and H3L_2-282 (Fig. 7e–i). Three Mpox patient sera did not bind to fragments which are shorter than H3L_2-184 but mainly recognized H3L_2-239 and H3L_2-282 (Fig. 7j–l). These findings suggest that the antibodies induced by MPXV infection with the similar specificity of mAb 3-3F (Fig. 3e) are the minority population in Mpox patient sera. In contrast, the major antibody profiles of Mpox patient sera are similar with the patterns recognized by mAbs 4-9F and 2-7G in the epitope mapping experiment (Fig. 3f & g).

## Discussion

Mpox can vary widely from a mild illness to a severe disease, potentially causing life-threatening complications and requiring intensive medical treatments in immunocompromised individuals (Mitja et al. 2023). The incubation period of Mpox can last up to 21 days, increasing the obstacle and difficulty in controlling the spread of infectious diseases (Titanji et al. 2024). In terms of Mpox diagnosis, polymerase chain reaction (PCR) can serve as a standard detection method (de Oliveira Thomasi et al. 2024; Fan et al. 2024). However, the operation of PCR needs a proper equipment, and it usually takes long processing time to obtain results, making it less suitable in remote areas with less medical resources. Anti-MPXV A29L antibodies have been applied in the development of the Mpox-specific antigen detection immunoassays (Davis et al. 2023; Liang et al. 2024; Sun et al. 2024; Yang et al. 2023; Ye et al. 2023). In our previous study, we showed that the LFIA rapid test using the mAb pairing of 2-5H and 3-8G can specifically detect MPXV A29L and distinguish it from VACV A27L (Liang et al. 2024). It has been reported that H3L is also an abundant surface antigen in the VACV proteome (Chung et al. 2006). In order to provide an alternative option for detection of MPXV while the existing rapid test for detecting A29L fails due to viral mutations, the anti-MPXV H3L mAbs were generated in the study. The 4-2A/3-3F-based LFIA rapid test can detect MPXV H3L protein and virion (Fig. 5). However, it must be noted that the mAbs 4-2A and 3-3F cannot distinguish between MPXV H3L and VACV H3L (Fig. 4).

The virus neutralizing activities in sera collected from MPXV-infected or vaccinated individuals who have received the third generation modified vaccinia Ankara (MVA)-based vaccine (IMVANEX) can be increased about 2.5-fold in the presence of complement, suggesting that complements play important roles in MPXV neutralization (Hubert et al. 2023). Studies focusing on VACV extracellular enveloped virion (EV) protein B5 showed that the protection against VACV EV infection provided by the IgG2a anti-B5 mAb B126 requires complement (Benhnia et al. 2009b). The depletion experiments, especially lacking of C3, exhibited a specific loss in the mAb B126-dependent protection (Cohen et al. 2011; Paran and Lustig 2010). Binding of human anti-B5 antibodies to VACV-infected cells potently directs complement-dependent cytotoxicity in an isotype-dependent manner (Benhnia et al. 2009a). In contrast, complement in combination with anti-A33 IgG did not neutralize VACV EV but induce complement-dependent virolysis of EV and thereby release the internal MV to be neutralized by the second IgG targeting the L1 membrane protein (Lustig et al. 2004). Interestingly, another study showed a different result that the human anti-A33 mAb VV22 with higher affinity developed by electrofusion generation of hybridomas using memory B cells from MVA-vaccinated donors and anti-B5 mAb h102 (Benhnia et al. 2009a) can neutralize EV in the presence of complement via opsonization of the EV particle surface (Benhnia et al. 2013). VACV MV contains surface proteins, such as A27L, H3L, and D8L, with main features for binding host cells and helping to establish an infection (Sagdat et al. 2024). Therefore, these surface proteins are expected to confer neutralization responses and become the major target antigens of neutralizing antibodies. Neutralization of VACV MV infection to Vero E6 cells by murine anti-H3L mAb clone 41 in the neutralization assay was enhanced in the presence of complement (Matho et al. 2012; McCausland et al. 2010). Neutralization of VACV MV by the anti-VACV D8 mAb LA5 was markedly increased from 20 to 80% in the presence of complement (Matho et al. 2012). Here, we also found that the neutralization of MPXV infection to Vero E6 cells by anti-MPXV H3L mAb 3-3F was significantly enhanced in the presence of complement (Fig. 6). However, the anti-MPXV H3L mAbs 4-2A, 1-1C, 3-8H, 4-9F, and 2-7G targeting binding epitopes, which are different from that of 3-3F (Fig. 3), were still lack of MPXV neutralizing activity in the presence of complement, indicating that the correct binding epitope recognized by the neutralizing antibody remains the key determinant and essential factor for the complement-dependent MPXV neutralization. It has been reported that the isotype of the murine neutralizing antibody shall be IgG2a, such as the anti-B5 mAb B126 (Benhnia et al. 2009a). Interestingly, the isotype of mAb 3-3F is IgG2b (Fig. 2b), but it still exhibited a complement-enhancing neutralizing activity. Recall that the isotype of 1-1C and 4-9F is IgG1 that may not effectively stimulate the antibody-dependent complement activity in vivo. Taken together, the roles of complement in enhancing the neutralizing activity of mAb 3-3F should be further characterized in the future. The in vivo toxicity, pharmacokinetics, or resistance potential of mAb 3-3F have not been evaluated, indicating that there is still a significant gap before clinical application.

It has been reported that the H3L epitope recognized by a fully human neutralizing antibody fh1A derived from a VACV-immune phage-display library is discontinuous and localized within the amino acid residues 15–19 and 232–237 (Khlusevich et al. 2018). Notably, the neutralizing epitope of fh1A does not match with the putative glycosaminoglycan binding sites located at amino acid residues 94–101 and 159–164 of H3L (Lin et al. 2000). It has been shown that most neutralization epitopes reside between 1 and 239 residues of H3L protein (Khlusevich et al. 2022). The amino acid residues necessary for binding with polyclonal neutralizing antibodies are localized on loops 13–34 and 231–239 of H3L protein (Khlusevich et al. 2022). Importantly, residues R16, P17, P18, E20, T21, V25, and A233 are crucial, and their substitutions led to the formation of epitopes unrecognizable by human anti-VACV polyclonal antibodies (Khlusevich et al. 2022). Here, we also found that the neutralizing binding epitope within residues 35–89 of H3L protein recognized by mAb 3-3F does not match with the putative glycosaminoglycan binding sites (Fig. 3e) and is also different from the neutralizing epitope of fh1A. The neutralizing mechanism of mAb 3-3F is probably caused by binding of mAb 3-3F to residues 35–89 of H3L protein that sterically interrupts virus adsorption to the host cell. It is also possible that mAb 3-3F can bind to an undefined glycosaminoglycan binding site. Alternatively, mAb 3-3F might neutralize virus infection by participating in the destabilization of the virus structure or aggregation of virions. The neutralizing mechanism mediated by mAb 3-3F should be further characterized by structural biology approach.

The strong antibody titer against H3L has been found after MPXV infection (Yang et al. 2024; Yefet et al. 2023). The seropositivity rate for anti-A29L and anti-H3L IgG during 1–7 days post symptom onset (d.p.o) could reach 86.36% and 79.55% and increased to 100% and 94.74% during 15–21 d.p.o, respectively (Yang et al. 2024). Therefore, A29L and H3L are considered the early diagnostic marker or the immune target for the identification of recent MPXV infection (Yang et al. 2024; Yefet et al. 2023). However, the serological study cannot provide more details on the antibody profiles induced by individual neutralizing epitopes. Thus, analysis of the antibody profiles against the major surface proteins of MPXV is very important for vaccine development and generation of neutralizing antibody. In our previous study, a series of truncated A29L fragments were utilized to analyze the antibody profiles of sera collected from Mpox patients (Liang et al. 2024). The results showed that Mpox patient sera contained significantly lower levels of antibodies targeting the N-terminal 2–34 residues of A29L. The large portions of antibodies induced by MPXV infection target the C-terminal region of A29L (Liang et al. 2024). Here, we also deployed the same approach using the truncated H3L fragments to analyze the anti-H3L antibody profiles of sera collected from Mpox patients who had not received the MVA-based vaccine before. We found that the responsive levels of H3L_2-34-specific antibodies were not observed in Mpox patient sera. In fact, the antibody levels for binding to H3L_2-89 are also very low (Fig. 7). Notably, the binding epitope of mAb 3-3F is located within the region of H3L_2-89 (Fig. 3e), indicating that the 3-3F-like neutralizing antibody was barely induced after MPXV infection. Thus, improvement of the immunogenicity of the H3L neutralizing epitope should be carefully taken into consideration while designing the MPXV vaccine containing H3L proteins for induction of potent neutralizing antibodies. Frankly, it should be mentioned that only ten patient sera were analyzed, which did not cover different disease stages or geographic origins. The limitation of the experimental results may potentially affect the generalizability of the conclusions. Furthermore, this study found that mAb 3-3F cannot completely inhibit MPXV infection to Vero E6 cells even in the presence of complement (Fig. 6c), implying that it should work in concert with other antibodies targeting additional MPXV surface proteins to achieve stronger neutralizing potency (Song et al. 2024; Tan et al. 2024).

Vaccines, diagnostic tools, and therapeutic agents are important countermeasures for preventing infectious disease (Kumari et al. 2022). WHO declared Mpox as a PHEIC for two times in 2 years regarding the accumulating cases reported in countries and regions where the MPXV had not been seen. In addition, the MPXV mutation strains continue to emerge with capability for sustained human-to-human transmission (Isidro et al. 2022; Vakaniaki et al. 2024). As we have learned from the experience of the COVID-19 pandemic, it will be behind the curve to develop the effective vaccines, reliable diagnostic methods, and therapeutic drugs when an unexpected Mpox outbreak with increasing severity and fatality rates suddenly appears and spreads globally. The studies on the neutralizing antibodies for inhibiting the functions of A29L and H3L can build up the foundation for developing the therapeutic agents against Mpox. If the LFIA rapid tests can combine diagnostics of A29L and H3L, it can also minimize the possible failure for detecting MPXV infection. Future work should make efforts on identifying more H3L neutralizing epitopes that may also contribute to design a better Mpox vaccine.

## Data Availability

All data associated with this study are included in the paper.
